# Rural and Urban Differences in Passenger-Vehicle–Occupant Deaths and Seat Belt Use Among Adults — United States, 2014

**DOI:** 10.15585/mmwr.ss6617a1

**Published:** 2017-09-22

**Authors:** Laurie F. Beck, Jonathan Downs, Mark R. Stevens, Erin K. Sauber-Schatz

**Affiliations:** 1 Division of Unintentional Injury Prevention, National Center for Injury Prevention and Control, CDC, Atlanta, Georgia; 2 Oak Ridge Institute for Science and Education, Oak Ridge, Tennessee; 3 Division of Analysis, Research, and Practice Integration, National Center for Injury Prevention and Control, CDC, Atlanta, Georgia

## Abstract

**Problem/Condition:**

Motor-vehicle crashes are a leading cause of death in the United States. Compared with urban residents, rural residents are at an increased risk for death from crashes and are less likely to wear seat belts. These differences have not been well described by levels of rurality.

**Reporting Period:**

2014.

**Description of Systems:**

Data from the Fatality Analysis Reporting System (FARS) and the Behavioral Risk Factor Surveillance System (BRFSS) were used to identify passenger-vehicle–occupant deaths from motor-vehicle crashes and estimate the prevalence of seat belt use. FARS, a census of U.S. motor-vehicle crashes involving one or more deaths, was used to identify passenger-vehicle–occupant deaths among adults aged ≥18 years. Passenger-vehicle occupants were defined as persons driving or riding in passenger cars, light trucks, vans, or sport utility vehicles. Death rates per 100,000 population, age-adjusted to the 2000 U.S. standard population and the proportion of occupants who were unrestrained at the time of the fatal crash, were calculated. BRFSS, an annual, state-based, random-digit–dialed telephone survey of the noninstitutionalized U.S. civilian population aged ≥18 years, was used to estimate prevalence of seat belt use. FARS and BRFSS data were analyzed by a six-level rural-urban designation, based on the U.S. Department of Agriculture 2013 rural-urban continuum codes, and stratified by census region and type of state seat belt enforcement law (primary or secondary).

**Results:**

Within each census region, age-adjusted passenger-vehicle–occupant death rates per 100,000 population increased with increasing rurality, from the most urban to the most rural counties: South, 6.8 to 29.2; Midwest, 5.3 to 25.8; West, 3.9 to 40.0; and Northeast, 3.5 to 10.8. (For the Northeast, data for the most rural counties were not reported because of suppression criteria; comparison is for the most urban to the second-most rural counties.) Similarly, the proportion of occupants who were unrestrained at the time of the fatal crash increased as rurality increased. Self-reported seat belt use in the United States decreased with increasing rurality, ranging from 88.8% in the most urban counties to 74.7% in the most rural counties. Similar differences in age-adjusted death rates and seat belt use were observed in states with primary and secondary seat belt enforcement laws.

**Interpretation:**

Rurality was associated with higher age-adjusted passenger-vehicle–occupant death rates, a higher proportion of unrestrained passenger-vehicle–occupant deaths, and lower seat belt use among adults in all census regions and regardless of state seat belt enforcement type.

**Public Health Actions:**

Seat belt use decreases and age-adjusted passenger-vehicle–occupant death rates increase with increasing levels of rurality. Improving seat belt use remains a critical strategy to reduce crash-related deaths in the United States, especially in rural areas where seat belt use is lower and age-adjusted death rates are higher than in urban areas. States and communities can consider using evidence-based interventions to reduce rural-urban disparities in seat belt use and passenger-vehicle–occupant death rates.

## Introduction

Motor-vehicle crashes are a leading cause of death nationwide, and persons traveling on rural roads or living in rural areas are at an increased risk for death from a motor-vehicle crash (*1*,*2*). In 2015, an estimated 19% of the U.S. population lived in rural areas, yet more than half (57%) of the 22,441 passenger-vehicle–occupant deaths occurred on rural roads (*3*,*4*). Although the total number of crashes is typically higher in urban areas, a much higher proportion of rural crashes result in death (*5*). Per 100 million vehicle miles traveled, the rate of all traffic deaths on rural roads (1.8) is 2.6 times the rate of all traffic deaths on urban roads (0.7) (*1*).

Multiple efforts in the United States are underway to reduce injuries and deaths from motor-vehicle crashes. In 2010, the U.S. Department of Health and Human Services established the *Healthy People 2020* objectives (https://www.healthypeople.gov/2020/topics-objectives). *Healthy People 2020* includes objectives to reduce all deaths related to motor-vehicle crashes to 12.4 deaths per 100,000 population and increase observed seat belt use to 92% by 2020. Also in 2010, CDC named prevention of motor-vehicle injuries a CDC winnable battle, a public health priority with a substantial impact on public health and with known effective strategies to address the issue (https://www.cdc.gov/winnablebattles/report/index.html). In October 2016, the National Safety Council, in partnership with the U.S. Department of Transportation, launched the Road to Zero initiative with the goal to eliminate road traffic deaths in the United States within 30 years (http://www.nsc.org/learn/NSC-Initiatives/Pages/The-Road-to-Zero.aspx). Increasing seat belt use, which reduces the risk for severe injury and death in a crash by approximately 50% (*6*), will help to reach these goals.

Part of the disparity in crash deaths in urban and rural areas is likely because of differences in seat belt use among passenger-vehicle occupants (*7*,*8*). Half (50%) of fatally injured occupants on rural roads were unrestrained in 2015, compared with 46% of fatally injured occupants on urban roads (*1*). Self-report surveys have documented lower levels of rural respondents who report always using a seat belt (*7*,*8*), even after adjusting for other factors associated with restraint use, such as body mass index (BMI), education, and household income (*7*).

One strategy states have used to increase seat belt use is to implement and enforce seat belt laws. Primary seat belt enforcement allows an officer to ticket drivers or passengers for not wearing a seat belt even if this is the only violation that has occurred. This differs from secondary seat belt enforcement, which only allows an officer to ticket a driver or passenger for failure to wear a seat belt when another violation (e.g., speeding or reckless driving) has also occurred. Primary enforcement, compared with secondary enforcement, has been associated with increased seat belt use (*7*,*9*) and reduced numbers of crash deaths (*10*,*11*).

Although rural-urban differences in risk behaviors and outcomes related to crash injuries are commonly reported using a dichotomous measure of rural versus urban (or nonmetropolitan versus metropolitan), other measures are available that allow examination of rural-urban differences in more detail (*12*,*13*). The purpose of this report is to examine differences in age-adjusted passenger-vehicle–occupant death rates, the proportion of passenger-vehicle occupants who were unrestrained at the time of the fatal crash, and self-reported seat belt use among adults in rural and urban counties, as measured on a six-level continuum and according to type of state seat belt enforcement. Alcohol-impaired driving and speeding among adults also were examined because these risk factors are related to lower rates of seat belt use (*14*). The findings in this report can be used by public health professionals, road safety professionals, and decision-makers to better understand and prevent rural-urban disparities in passenger-vehicle–occupant deaths and seat belt use to prevent injuries and deaths.

## Methods

### Data Sources and Analyses 

The prevalence of self-reported seat belt use and alcohol-impaired driving was determined for the United States (50 states and the District of Columbia [DC]) using 2014 data (the most recent available) from CDC’s Behavioral Risk Factor Surveillance System (BRFSS). BRFSS is an annual, random-digit–dialed telephone survey of the noninstitutionalized U.S. civilian population aged ≥18 years conducted annually in all 50 states, DC, Guam, Puerto Rico, and the U.S. Virgin Islands. Detailed information regarding the survey is available online (https://www.cdc.gov/brfss/annual_data/annual_2014.html). The median response rate for the 50 states and DC in 2014 was 47.0%; response rates ranged from 25.1% (California) to 60.1% (South Dakota) (https://www.cdc.gov/brfss/annual_data/2014/pdf/2014_dqr.pdf). The response rate is the number of respondents who completed the survey as a proportion of all eligible and likely eligible persons. Statistical software that accounts for the complex sampling design of BRFSS was used for the analyses. Prevalence estimates and their 95% confidence intervals (CIs), rate ratios (RRs) and their 95% CIs, and p values comparing primary and secondary state enforcement types were calculated, and tests for trends in seat belt use across the rural-urban continuum were conducted by modeling the county classification as a continuous variable. Results with p<0.05 were considered statistically significant. Estimates based on <20 respondents or with a relative standard error (RSE) ≥30% were deemed unreliable and were suppressed. Estimates based on 20–49 respondents and RSE <30% were reported but noted as potentially unreliable.

The age-adjusted passenger-vehicle–occupant death rates among adults aged ≥18 years and proportion of adult passenger-vehicle occupants who were unrestrained at the time of the fatal crash were calculated for the United States (50 states and DC) using data from the Fatality Analysis Reporting System (FARS) 2014 Final FARS Report File (the most recent available) (*15*). FARS is a census of all U.S. crashes on public roadways involving one or more deaths (that occur within 30 days of the crash) that is maintained by the National Highway Traffic Safety Administration (NHTSA) through a cooperative agreement with all states, DC, and Puerto Rico (https://crashstats.nhtsa.dot.gov/Api/Public/ViewPublication/811992). 

Death rates are age-adjusted to the 2000 U.S. standard population. Age-adjusted rates are reported per 100,000 population, and 95% CIs were constructed using a gamma distribution. To calculate age-adjusted passenger-vehicle–occupant death rates, statistical software was used to aggregate data by age group, seat belt enforcement type, and county where the crash occurred. Next, 2014 bridged-race population estimates for the U.S. population aged ≥18 years were obtained from the National Center for Health Statistics (NCHS) vintage 2015 bridged-race postcensal population estimates (*16*). This data set provides single year-of-age population estimates at the county level by year. Aggregated death data from FARS were matched with census population data and county classification using Federal Information Processing Standards codes. Because motor-vehicle death rates have been previously shown to vary by census region, (*17*) and seat belt enforcement type also varies by census region, age-adjusted death rates were stratified by census region. Age-adjusted death rates also were calculated by whether the crash death was speeding related or involved an alcohol-impaired driver. 

To determine the proportion of passenger-vehicle occupants who were unrestrained at the time of the fatal crash, FARS data for all fatally injured passenger-vehicle occupants aged ≥18 years were grouped by county classification and state seat belt enforcement type. Among the 19,528 deaths meeting the original study criteria, 1,542 (7.9%) were excluded because restraint use at the time of the crash was unknown. 

Age-adjusted death rate RRs and 95% CIs were used to assess whether death rates differed significantly by enforcement type. Age-adjusted death rates based on <20 deaths or with RSE ≥30% were deemed unreliable and suppressed. Age-adjusted alcohol-impaired driving death rates were pooled and analyzed using the appropriate statistical procedures for multiply imputed data. Joinpoint regression was used to assess linear trends both in age-adjusted death rates and proportion of deaths among unrestrained passengers across the rural-urban continuum. Zero joinpoints were used to assess a general linear trend, and significance was assessed at the 0.05 level.

### Rural-Urban Continuum and Metropolitan Status

The U.S. Department of Agriculture (USDA) developed the 2013 rural-urban continuum codes (RUCCs), which classify counties into one of nine levels (*13*). Counties are first identified as metropolitan or nonmetropolitan using Office of Management and Budget designations. Metropolitan counties are further classified according to the population size of the larger metropolitan area to which they belong. Nonmetropolitan counties are classified according to the urban population size within the county as well as their adjacency to a metropolitan county.

In this report, USDA’s nine RUCCs were collapsed into six levels, based on the population of the largest urban area and metropolitan or nonmetropolitan status: RUCC 1 (metropolitan areas of ≥1,000,000 population), RUCC 2 (metropolitan areas of 250,000–999,999 population), RUCC 3 (metropolitan areas of <250,000 population), RUCCs 4 and 5 (nonmetropolitan areas with an urban population of ≥20,000), RUCCs 6 and 7 (nonmetropolitan areas with an urban population of 2,500–19,999), and RUCCs 8 and 9 (nonmetropolitan areas with an urban population of <2,500). In this report, the terms urban and rural are used to refer to counties along a continuum based on urban population size: more rural counties have a smaller urban population, and more urban counties have a larger urban population. The term metropolitan is used when referencing results for the three metropolitan areas combined, and the term nonmetropolitan is used when referencing results for the three nonmetropolitan areas combined. This terminology is consistent with the USDA RUCC definitions (*13*).

### Seat Belt Enforcement

Seat belt enforcement type varies by state and can be primary or secondary. In states with primary enforcement laws, officers can ticket a driver or passenger for not wearing a seat belt, even if no other offense has occurred. In states with secondary laws, officers can only ticket drivers or passengers for not wearing a seat belt when another traffic offense has occurred. In 2014, a total of 33 states and DC had primary enforcement, 16 states had secondary enforcement, and one state (New Hampshire) had no requirement for seat belt use by adults (*18*). States with primary enforcement in 2014 included Alabama, Alaska, Arkansas, California, Connecticut, Delaware, DC, Florida, Georgia, Hawaii, Illinois, Indiana, Iowa, Kansas, Kentucky, Louisiana, Maine, Maryland, Michigan, Minnesota, Mississippi, New Jersey, New Mexico, New York, North Carolina, Oklahoma, Oregon, Rhode Island, South Carolina, Tennessee, Texas, Washington, West Virginia, and Wisconsin. States with secondary enforcement in 2014 included Arizona, Colorado, Idaho, Massachusetts, Missouri, Montana, Nebraska, Nevada, North Dakota, Ohio, Pennsylvania, South Dakota, Utah, Vermont, Virginia, and Wyoming. New Hampshire was grouped with secondary enforcement states for analysis purposes. Distribution of primary and secondary enforcement provisions differed by census region (Northeast, South, Midwest, and West) (*19*). In the South, 15 states and DC (94%) had primary enforcement, and only one state (Virginia) had secondary enforcement. In the Midwest, seven states (58%) had primary enforcement and five states had secondary enforcement. In the Northeast, five states (56%) had primary enforcement, three states had secondary enforcement, and one state (New Hampshire) had no enforcement. In the West, six states (46%) had primary enforcement, and seven states had secondary enforcement.

### Passenger-Vehicle–Occupant Deaths 

FARS data for passenger-vehicle–occupant deaths among adults aged ≥18 years were included in this report. Passenger-vehicle occupants are defined as persons driving or riding in passenger cars, light trucks, vans, or sport utility vehicles. Deaths were limited to those among passenger-vehicle occupants aged ≥18 years because certain commercial-vehicle occupants might have been covered by different seat belt requirements and certain states have separate seat belt enforcement requirements for those aged <18 years (*20*). Of the 32,744 motor vehicle crash deaths in 2014, a total of 19,528 met criteria for inclusion in this report. Additional elements in FARS included county of the crash location, whether a fatal crash involved speeding (e.g., exceeding the posted speed limit or driving too fast for conditions) or an alcohol-impaired driver (driver with blood alcohol concentration ≥0.08 g/dL). FARS uses multiple imputation when blood alcohol test results are not reported. One death did not have a recorded county; the most likely county was determined using the latitude and longitude coordinates of the crash.

### Self-Reported Seat Belt Use 

To assess seat belt use, BRFSS survey respondents were asked, “How often do you use seat belts when you drive or ride in a car?” Response options were “always,” “nearly always,” “sometimes,” “seldom,” or “never.” Seat belt use was defined as always wearing a seat belt. Survey respondents who said they did not drive or ride in a car (0.2%) or who were coded as “do not know,” “refused,” or “missing” (5.8%) were excluded. The BRFSS analyses compared seat belt use (i.e., always wears a seat belt) between primary and secondary state enforcement types across the six-level rural-urban designation (based on county of residence) and selected demographic, health-related, and behavioral characteristics. Each of the six rural-urban classifications were stratified by census region. Selected self-reported characteristics (sex, race/ethnicity, age, education, marital status, employment status, BMI, and alcohol-impaired driving) were categorized according to whether counties were metropolitan or nonmetropolitan. To assess alcohol-impaired driving, BRFSS survey respondents who reported that they had had at least one alcoholic drink in the past 30 days were asked, “During the past 30 days, how many times have you driven when you’ve had perhaps too much to drink?” Self-reported alcohol-impaired driving was categorized as driving after having too much to drink at least once in the past 30 days. Survey respondents were excluded from alcohol-impaired driving analyses if they were coded as “do not know,” “refused,” or “missing” (0.2%).

## Results

### Passenger-Vehicle–Occupant Deaths

Within each census region, age-adjusted passenger-vehicle–occupant death rates were significantly higher in nonmetropolitan counties than in metropolitan counties (Table 1), both overall and in primary and secondary enforcement states. For example, in the Northeast, age-adjusted death rates per 100,000 population were 4.2 in metropolitan counties and 9.6 in nonmetropolitan counties. In northeastern states with primary enforcement, age-adjusted death rates per 100,000 population were 3.5 in metropolitan counties and 9.2 in nonmetropolitan counties. In northeastern states with secondary enforcement, age-adjusted death rates per 100,000 population were 5.4 in metropolitan counties and 10.0 in nonmetropolitan counties.

**TABLE 1:** Number of passenger-vehicle–occupant deaths and age-adjusted death rates per 100,000 population among adults aged ≥18 years, by census region, rural-urban designation, and type of state seat belt enforcement — Fatality Analysis Reporting System, United States, 2014

Census region and rural-urban designation^†^	Total		Primary seat belt enforcement		Secondary seat belt enforcement*		
	No. of deaths	Age-adjusted death rate (95% CI)	No. of deaths	Age-adjusted death rate (95% CI)	No. of deaths	Age-adjusted death rate (95% CI)	Secondary-primary RR (95% CI)
**Northeast**	**2,066**	**4.7 (4.5–4.9)**	**1,055**	**3.9 (3.7–4.1)**	**1,011**	**5.9 (5.6–6.3)**	**1.52 (1.40–1.66)^§^**
Metropolitan counties, overall	**1,709**	**4.2 (4.0–4.4)**	900	3.5 (3.3–3.8)	809	5.4 (5.0–5.8)	1.52 (1.38–1.67)^§^
In metropolitan area with ≥1,000,000 population	**1,017**	**3.5 (3.3–3.7)**	612	3.0 (2.8–3.3)	405	4.4 (4.0–4.9)	1.45 (1.27–1.64)^§^
In metropolitan area with 250,000–999,999 population	**514**	**5.9 (5.4–6.5)**	220	5.2 (4.5–5.9)	294	6.7 (5.9–7.5)	1.29 (1.08–1.55)^§^
In metropolitan area with <250,000 population	**178**	**7.5 (6.4–8.8)**	68	7.1 (5.5–9.1)	110	7.8 (6.4–9.5)	1.10 (0.81–1.50)
Nonmetropolitan counties, overall	**357**	**9.6 (8.6–10.7)**	155	9.2 (7.7–10.8)	202	10.0 (8.6–11.5)	1.09 (0.88–1.36)
Urban population of ≥20,000	**177**	**8.5 (7.2–9.9)**	67	7.5 (5.8–9.6)	110	9.2 (7.5–11.2)	1.23 (0.90–1.68)
Urban population of 2,500–19,999	**161**	**10.8 (9.1–12.7)**	79	10.7 (8.4–13.5)	82	10.8 (8.5–13.6)	1.01 (0.73–1.40)
Completely rural or <2,500 urban population	—^¶^	—	—	—	—	—	—
**South**	**9,720**	**10.7 (10.5–10.9)**	**9,268**	**10.9 (10.7–11.2)**	**452**	**7.0 (6.4–7.7)**	**0.64 (0.58–0.71)^§^**
Metropolitan counties, overall	**6,459**	**8.5 (8.3–8.7)**	6,143	8.7 (8.5–9.0)	316	5.7 (5.1–6.3)	0.65 (0.58–0.73)^§^
In metropolitan area with ≥1,000,000 population	**3,089**	**6.8 (6.6–7.1)**	2,886	7.1 (6.8–7.3)	203	4.6 (4.0–5.3)	0.66 (0.57–0.76)^§^
In metropolitan area with 250,000–999,999 population	**2,250**	**10.5 (10.0–10.9)**	2,207	10.5 (10.1–11.0)	—	—	—
In metropolitan area with <250,000 population	**1,120**	**12.7 (12.0–13.5)**	1,050	12.8 (12.0–13.6)	70	11.3 (8.8–14.4)	0.88 (0.69–1.13)
Nonmetropolitan counties, overall	**3,261**	**21.9 (21.1–22.7)**	3,125	22.2 (21.4–23.0)	136	16.9 (14.1–20.1)	0.76 (0.64–0.91)^§^
Urban population of ≥20,000	**829**	**16.5 (15.4–17.7)**	803	16.5 (15.4–17.7)	—	—	—
Urban population of 2,500–19,999	**1,920**	**23.8 (22.7–24.9)**	1,840	24.0 (22.9–25.2)	80	19.4 (15.2–24.3)	0.81 (0.64–1.02)
Completely rural or <2,500 urban population	**512**	**29.2 (26.7–32.0)**	482	31.5 (28.6–34.5)	—	—	—
**Midwest**	**4,197**	**8.1 (7.9–8.4)**	**2,659**	**7.5 (7.2–7.8)**	**1,538**	**9.6 (9.1–10.1)**	**1.28 (1.20–1.37)^§^**
Metropolitan counties, overall	**2,465**	**6.2 (5.9–6.4)**	1,656	5.9 (5.6–6.2)	809	6.8 (6.3–7.2)	1.14 (1.05–1.25)^§^
In metropolitan area with ≥1,000,000 population	**1,271**	**5.3 (5.0–5.6)**	856	5.0 (4.7–5.4)	415	6.1 (5.6–6.8)	1.22 (1.09–1.38)^§^
In metropolitan area with 250,000–999,999 population	**657**	**7.0 (6.5–7.6)**	412	7.1 (6.4–7.8)	245	6.9 (6.1–7.9)	0.98 (0.83–1.15)
In metropolitan area with <250,000 population	**537**	**8.0 (7.3–8.7)**	388	7.6 (6.8–8.4)	149	9.1 (7.7–10.7)	1.20 (0.99–1.46)
Nonmetropolitan counties, overall	**1,732**	**15.2 (14.5–16.0)**	1,003	13.6 (12.7–14.5)	729	18.1 (16.8–19.5)	1.33 (1.21–1.47)^§^
Urban population of ≥20,000	**520**	**11.6 (10.6–12.7)**	287	10.6 (9.4–12.0)	233	13.0 (11.4–14.8)	1.22 (1.03–1.46)^§^
Urban population of 2,500–19,999	**900**	**16.0 (14.9–17.1)**	593	14.9 (13.7–16.2)	307	18.4 (16.3–20.6)	1.23 (1.07–1.42)^§^
Completely rural or <2,500 urban population	**312**	**25.8 (22.8–28.9)**	123	18.5 (15.2–22.2)	189	34.8 (29.8–40.3)	1.88 (1.48–2.39)^§^
**West**	**3,545**	**6.2 (6.0–6.4)**	**2,282**	**5.5 (5.3–5.8)**	**1,263**	**8.0 (7.5–8.4)**	**1.44 (1.35–1.54)^§^**
Metropolitan counties, overall	**2,654**	**5.1 (4.9–5.3)**	1,877	4.9 (4.6–5.1)	777	5.8 (5.4–6.2)	1.19 (1.10–1.30)^§^
In metropolitan area with ≥1,000,000 population	**1,382**	**3.9 (3.7–4.2)**	1,020	3.7 (3.5–4.0)	362	4.7 (4.2–5.2)	1.25 (1.11–1.41)^§^
In metropolitan area with 250,000–999,999 population	**787**	**6.4 (6.0–6.9)**	579	6.7 (6.2–7.3)	208	5.7 (4.9–6.5)	0.84 (0.72–0.98)^§^
In metropolitan area with <250,000 population	**485**	**10.7 (9.7–11.7)**	278	10.5 (9.3–11.9)	207	10.9 (9.4–12.5)	1.03 (0.86–1.24)
Nonmetropolitan counties, overall	**891**	**18.0 (16.9–19.3)**	405	15.8 (14.3–17.5)	486	20.4 (18.6–22.3)	1.29 (1.12–1.47)^§^
Urban population of ≥20,000	**396**	**14.9 (13.5–16.5)**	235	14.7 (12.8–16.7)	161	15.4 (13.0–17.9)	1.05 (0.85–1.28)
Urban population of 2,500–19,999	**358**	**18.5 (16.6–20.6)**	133	16.1 (13.4–19.2)	225	20.1 (17.5–23.0)	1.25 (1.00–1.56)
Completely rural or <2,500 urban population	**137**	**40.0 (33.3–47.6)**	—	—	100	47.4 (38.2–58.1)	—

**Abbreviations:** CI = confidence interval; RR = rate ratio.

* In 2014, a total of 33 states and the District of Columbia (DC) had primary enforcement, 16 states had secondary enforcement, and one state (New Hampshire) had no requirement for seat belt use by adults. States with primary enforcement in 2014 included Alabama, Alaska, Arkansas, California, Connecticut, Delaware, DC, Florida, Georgia, Hawaii, Illinois, Indiana, Iowa, Kansas, Kentucky, Louisiana, Maine, Maryland, Michigan, Minnesota, Mississippi, New Jersey, New Mexico, New York, North Carolina, Oklahoma, Oregon, Rhode Island, South Carolina, Tennessee, Texas, Washington, West Virginia, and Wisconsin. States with secondary enforcement in 2014 included Arizona, Colorado, Idaho, Massachusetts, Missouri, Montana, Nebraska, Nevada, North Dakota, Ohio, Pennsylvania, South Dakota, Utah, Vermont, Virginia, and Wyoming. New Hampshire was grouped with secondary enforcement states for analysis purposes.

^†^ Census regions were determined using the U.S. Census Bureau definition (https://www2.census.gov/geo/pdfs/maps-data/maps/reference/us_regdiv.pdf). Rural and urban designations were determined using the U.S. Department of Agriculture’s 2013 rural-urban continuum codes (https://www.ers.usda.gov/data-products/rural-urban-continuum-codes). Metropolitan counties include counties in metropolitan areas of ≥1,000,000 population, counties in metropolitan areas of 250,000–999,999 population, and counties in metropolitan areas of <250,000 population, Nonmetropolitan counties include counties in nonmetropolitan areas with an urban population of ≥20,000, counties in nonmetropolitan areas with an urban population of 2,500–19,999, and counties that are completely rural or with <2,500 urban population.

^§^ Age-adjusted RRs comparing rates in secondary enforcement states to rates in primary enforcement states are significantly different from 1 (p<0.05).

^¶^ Estimates based on <20 respondents or with RSE ≥30% were deemed unreliable and were suppressed.

Also in each census region, age-adjusted passenger-vehicle–occupant death rates generally increased with increasing rurality (Table 1). In the Northeast, age-adjusted death rates per 100,000 population increased from 3.5 in the most urban counties to 10.8 in the second-most rural counties (joinpoint regression test for trend, p<0.001). Results for the most rural counties in the Northeast were suppressed because they had <20 respondents or RSE ≥30%. In the South, age-adjusted death rates per 100,000 population increased from 6.8 in the most urban counties to 29.2 in the most rural counties (test for trend, p<0.001). In the Midwest, age-adjusted death rates per 100,000 population increased from 5.3 in the most urban counties to 25.8 in the most rural counties (test for trend, p<0.01). In the West, age-adjusted death rates per 100,000 population increased from 3.9 in the most urban counties to 40.0 in the most rural counties (test for trend, p<0.01). Similarly, age-adjusted passenger-vehicle–occupant death rates increased with increasing rurality among states with primary enforcement and among states with secondary enforcement (Table 1).

RRs were calculated to assess the association of enforcement type (primary or secondary) and age-adjusted passenger-vehicle–occupant death rates. These relationships were not consistent across all census regions or county classifications (Table 1). Overall, age-adjusted passenger-vehicle–occupant death rates in the Northeast, Midwest, and West were significantly higher in secondary enforcement states than in primary enforcement states (RR = 1.52, RR = 1.28, RR = 1.44, respectively). However, in the South, where only one of 17 states had secondary enforcement, age-adjusted passenger-vehicle–occupant death rates were significantly higher in the primary enforcement states than in the one secondary enforcement state (RR = 0.64). Similar relationships within each census region were observed for many of the rural-urban levels, although not all associations were statistically significant (Table 1).

**FIGURE 1:**
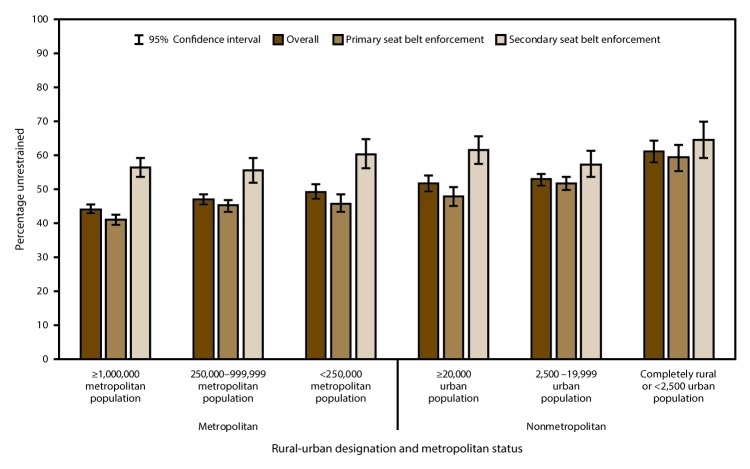
Percentage of passenger-vehicle occupants who were unrestrained at time of fatal crash, among adults aged ≥18 years, by rural-urban designation, metropolitan status,* and type of state seat belt enforcement^†^ — Fatality Analysis Reporting System, United States, 2014 * Rural and urban designations were determined using the U.S. Department of Agriculture’s 2013 rural-urban continuum codes. Metropolitan counties include counties in metropolitan areas of ≥1,000,000 population, counties in metropolitan areas of 250,000–999,999 population, and counties in metropolitan areas of <250,000 population. Nonmetropolitan counties include counties in nonmetropolitan areas with an urban population of ≥20,000, counties in nonmetropolitan areas with an urban population of 2,500–19,999, and counties that are completely rural or with <2,500 urban population. ^†^ Primary or secondary enforcement. In 2014, a total of 33 states and the District of Columbia (DC) had primary enforcement, 16 states had secondary enforcement, and one state (New Hampshire) had no requirement for seat belt use by adults. States with primary enforcement in 2014 included Alabama, Alaska, Arkansas, California, Connecticut, Delaware, DC, Florida, Georgia, Hawaii, Illinois, Indiana, Iowa, Kansas, Kentucky, Louisiana, Maine, Maryland, Michigan, Minnesota, Mississippi, New Jersey, New Mexico, New York, North Carolina, Oklahoma, Oregon, Rhode Island, South Carolina, Tennessee, Texas, Washington, West Virginia, and Wisconsin. States with secondary enforcement in 2014 included Arizona, Colorado, Idaho, Massachusetts, Missouri, Montana, Nebraska, Nevada, North Dakota, Ohio, Pennsylvania, South Dakota, Utah, Vermont, Virginia, and Wyoming. New Hampshire was grouped with secondary enforcement states for analysis purposes.

The proportions of passenger-vehicle occupants who were unrestrained at the time of the fatal crash also were examined by rurality and enforcement type (Figure 1). The proportion of passenger-vehicle occupants who were unrestrained at the time of the fatal crash increased as rurality increased, overall and in both primary and secondary enforcement states (joinpoint regression test for linear trend: overall, p<0.01; primary states, p<0.01; secondary states, p<0.05). In primary enforcement states, the proportion of unrestrained passenger-vehicle–occupant deaths was 41.3% in the most urban counties, compared with 59.5% in the most rural counties. In secondary enforcement states, these proportions were 56.7% in the most urban counties and 64.7% in the most rural counties (Figure 1).

In the United States, the age-adjusted speeding-associated passenger-vehicle–occupant death rate per 100,000 population was 2.2 in metropolitan counties and 5.5 in nonmetropolitan counties (data not shown). Similarly, age-adjusted speeding-associated passenger-vehicle–occupant death rates were 2.3–3.1 times higher in nonmetropolitan counties compared with metropolitan counties across the four census regions. In most census regions, age-adjusted speeding-related passenger-vehicle–occupant death rates were significantly higher in secondary enforcement states than in primary enforcement states. However, in the South, age-adjusted speeding-related passenger-vehicle–occupant death rates were significantly lower in the one secondary enforcement state than in primary enforcement states, both in metropolitan counties (RR = 0.38) and nonmetropolitan counties (RR = 0.49).

Age-adjusted passenger-vehicle–occupant death rates for crashes involving alcohol-impaired driving were higher in nonmetropolitan counties than in metropolitan counties in the United States overall (6.2 and 2.4 deaths per 100,000 population, respectively; data not shown) and in each of the four census regions. Similar to the speeding-associated deaths, age-adjusted death rates that involved alcohol-impaired driving were higher in secondary enforcement states than in primary enforcement states in every census region except the South. In the South, age-adjusted passenger-vehicle–occupant death rates that involved alcohol-impaired driving were lower in the one secondary enforcement state (2.1 and 5.3 deaths per 100,000 population in metropolitan and nonmetropolitan counties, respectively) than in primary enforcement states (3.5 and 7.8 deaths per 100,000 population in metropolitan and nonmetropolitan counties, respectively).

### Self-Reported Seat Belt Use

**FIGURE 2:**
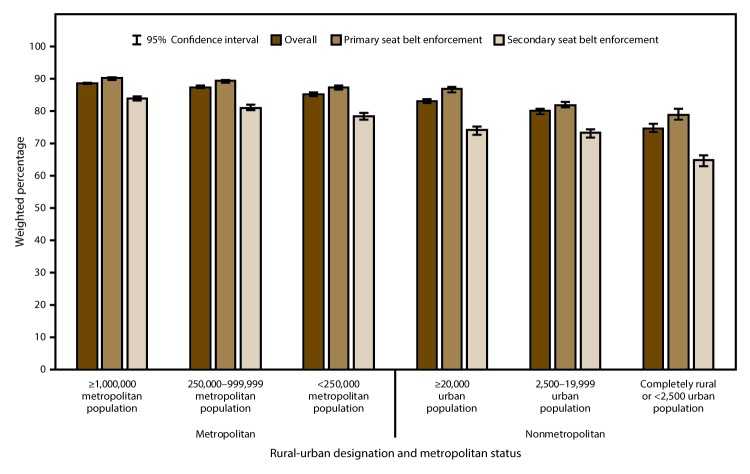
Self-reported seat belt use among adults aged ≥18 years, by rural-urban designation, metropolitan status,* and type of state seat belt enforcement^†^ — Behavioral Risk Factor Surveillance System, United States, 2014 * Rural and urban designations were determined using the U.S. Department of Agriculture’s 2013 rural-urban continuum codes. Metropolitan counties include counties in metropolitan areas of ≥1,000,000 population, counties in metropolitan areas of 250,000–999,999 population, and counties in metropolitan areas of <250,000 population. Nonmetropolitan counties include counties in nonmetropolitan areas with an urban population of ≥20,000, counties in nonmetropolitan areas with an urban population of 2,500–19,999, and counties that are completely rural or with <2,500 urban population. ^†^ Primary or secondary enforcement. In 2014, a total of 33 states and the District of Columbia (DC) had primary enforcement, 16 states had secondary enforcement, and one state (New Hampshire) had no requirement for seat belt use by adults. States with primary enforcement in 2014 included Alabama, Alaska, Arkansas, California, Connecticut, Delaware, DC, Florida, Georgia, Hawaii, Illinois, Indiana, Iowa, Kansas, Kentucky, Louisiana, Maine, Maryland, Michigan, Minnesota, Mississippi, New Jersey, New Mexico, New York, North Carolina, Oklahoma, Oregon, Rhode Island, South Carolina, Tennessee, Texas, Washington, West Virginia, and Wisconsin. States with secondary enforcement in 2014 included Arizona, Colorado, Idaho, Massachusetts, Missouri, Montana, Nebraska, Nevada, North Dakota, Ohio, Pennsylvania, South Dakota, Utah, Vermont, Virginia, and Wyoming. New Hampshire was grouped with secondary enforcement states for analysis purposes.

In 2014, self-reported seat belt use (i.e., respondent always wears a seat belt) in the United States was 86.9% overall (Table 2). Seat belt use was highest in the most urban counties (88.8%) and lowest in the most rural counties (74.7%), and the trend of decreasing seat belt use with increasing rurality was significant (Figure 2). Similar patterns were observed in both the primary and secondary enforcement states. Seat belt use was highest in the West (90.1%), followed by the South (88.2%), the Northeast (84.0%), and the Midwest (83.7%) (Table 2). Within each region, seat belt use also decreased significantly with increasing rurality (Supplementary Table, https://stacks.cdc.gov/view/cdc/47557).

**TABLE 2:** Self-reported seat belt use among adults aged ≥18 years, by rural-urban designation, census region, and type of state seat belt enforcement — Behavioral Risk Factor Surveillance System, United States, 2014

Rural-urban designation and census region	Seat belt use (always wears)				
	Total		Primary seat belt enforcement	Secondary seat belt enforcement*	Primary-secondary RR (95% CI)
	No.	Weighted % (95% CI)	Weighted % (95% CI)	Weighted % (95% CI)	
**United States**	**429,058**	**86.9 (86.6–87.1)**	**88.7 (88.5–89.0)**	**80.7 (80.3–81.1)**	**1.10 (1.09–1.11)^§^**
**Rural-urban designation^†^**					
Metropolitan counties, overall	**298,677**	**88.1 (87.9–88.3)**	89.7 (89.5–90.0)	82.5 (82.1–83.0)	1.09 (1.08–1.09)^§^
In metropolitan area with ≥1,000,000 population	**150,796**	**88.8 (88.5–89.1)**	90.2 (89.8–90.6)	83.9 (83.3–84.5)	1.08 (1.07–1.08)^§^
In metropolitan area with 250,000–999,999 population	**89,056**	**87.6 (87.1–88.0)**	89.5 (89.0–89.9)	81.2 (80.3–82.0)	1.10 (1.09–1.12)^§^
In metropolitan area with <250,000 population	**58,825**	**85.3 (84.7–85.9)**	87.5 (86.8–88.2)	78.5 (77.4–79.6)	1.11 (1.10–1.13)^§^
Nonmetropolitan counties, overall	**130,324**	**80.6 (80.1–81.0)**	83.5 (82.9–84.0)	72.5 (71.7–73.3)	1.15 (1.14–1.17)^§^
Urban population of ≥20,000	**42,775**	**83.2 (82.4–83.9)**	86.9 (86.0–87.7)	74.2 (72.7–75.5)	1.17 (1.15–1.20)^§^
Urban population of 2,500–19,999	**64,047**	**80.0 (79.3–80.7)**	82.0 (81.2–82.8)	73.3 (72.1–74.5)	1.12 (1.10–1.14)^§^
Completely rural or <2,500 urban population	**23,502**	**74.7 (73.4–76.0)**	79.1 (77.3–80.7)	64.7 (62.8–66.6)	1.22 (1.18–1.27)^§^
**Census region** ^¶^					
Northeast	**76,968**	**84.0 (83.4–84.5)**	87.7 (87.0–88.4)	78.1 (77.3–78.9)	1.12 (1.11–1.14)^§^
South	**128,702**	**88.2 (87.9–88.6)**	88.3 (88.0–88.7)	87.2 (86.1–88.1)	1.01 (1.00–1.03)^§^
Midwest	**120,363**	**83.7 (83.3–84.1)**	86.2 (85.7–86.7)	78.3 (77.5–79.1)	1.10 (1.09–1.11)^§^
West	**103,025**	**90.1 (89.6–90.5)**	93.1 (92.5–93.7)	83.2 (82.7–83.7)	1.12 (1.11–1.13)^§^

**Abbreviations:** CI = confidence interval; RR = rate ratio.

* In 2014, a total of 33 states and the District of Columbia (DC) had primary enforcement, 16 states had secondary enforcement, and one state (New Hampshire) had no requirement for seat belt use by adults. States with primary enforcement in 2014 included Alabama, Alaska, Arkansas, California, Connecticut, Delaware, DC, Florida, Georgia, Hawaii, Illinois, Indiana, Iowa, Kansas, Kentucky, Louisiana, Maine, Maryland, Michigan, Minnesota, Mississippi, New Jersey, New Mexico, New York, North Carolina, Oklahoma, Oregon, Rhode Island, South Carolina, Tennessee, Texas, Washington, West Virginia, and Wisconsin. States with secondary enforcement in 2014 included Arizona, Colorado, Idaho, Massachusetts, Missouri, Montana, Nebraska, Nevada, North Dakota, Ohio, Pennsylvania, South Dakota, Utah, Vermont, Virginia, and Wyoming. New Hampshire was grouped with secondary enforcement states for analysis purposes.

^†^ Rural and urban designations were determined using the U.S. Department of Agriculture’s 2013 rural-urban continuum codes (https://www.ers.usda.gov/data-products/rural-urban-continuum-codes). Metropolitan counties include counties in metropolitan areas of ≥1,000,000 population, counties in metropolitan areas of 250,000–999,999 population, and counties in metropolitan areas of <250,000 population, Nonmetropolitan counties include counties in nonmetropolitan areas with an urban population of ≥20,000, counties in nonmetropolitan areas with an urban population of 2,500–19,999, and counties that are completely rural or with <2,500 urban population.

^§^ RRs comparing seat belt use in primary enforcement states to seat belt use in secondary enforcement states are significantly different from 1 (p<0.05).

^¶^ Census regions were determined using the U.S. Census Bureau definition (https://www2.census.gov/geo/pdfs/maps-data/maps/reference/us_regdiv.pdf).

Overall, for all rural-urban designations and all census regions, seat belt use was significantly higher in primary enforcement states than in secondary enforcement states (Table 2). Similar rural-urban patterns in seat belt use by enforcement type were observed within each census region (Supplementary Table, https://stacks.cdc.gov/view/cdc/47557), with the exception of the South. Other than the South overall, the southern metropolitan area overall, and southern counties with an urban population of ≥20,000, no statistically significant differences were found in seat belt use between primary enforcement states and the one secondary enforcement state in the South.

Seat belt use also was examined by metropolitan status and selected demographic, health-related, and behavioral characteristics (Table 3). For almost all characteristics examined, seat belt use was significantly higher in metropolitan counties than in nonmetropolitan counties. For example, seat belt use was 84.9% among men and 91.1% among women in metropolitan counties, compared with 74.4% among men and 86.4% among women in nonmetropolitan counties. Seat belt use among those who reported alcohol-impaired driving in the past 30 days was 73.7% in metropolitan counties, compared with 60.6% in nonmetropolitan counties.

**TABLE 3:** Self-reported seat belt use among adults aged ≥18 years, by metropolitan status, type of state seat belt enforcement, and selected characteristics — Behavioral Risk Factor Surveillance System, United States, 2014

Selected characteristics	Seat belt use (always wears)				
	Total		Primary seat belt enforcement	Secondary seat belt enforcement*	Primary-secondary RR (95% CI)
	No.	Weighted % (95% CI)	Weighted % (95% CI)	Weighted % (95% CI)	
**Metropolitan counties, overall**	**298,677**	**88.1 (87.9–88.3)**	**89.7 (89.5–90.0)**	**82.5 (82.1–83.0)**	**1.09 (1.08–1.09)^†^**
**Sex**					
Male	**124,325**	**84.9 (84.5–85.2)**	87.0 (86.5–87.4)	77.8 (77.1–78.5)	1.12 (1.11–1.13)^†^
Female	**174,352**	**91.1 (90.8–91.4)**	92.3 (92.0–92.7)	86.9 (86.4–87.5)	1.06 (1.05–1.07)^†^
**Race/Ethnicity**					
White, non-Hispanic	**227,998**	**88.1 (87.8–88.3)**	90.0 (89.7–90.3)	82.7 (82.2–83.2)	1.09 (1.08–1.10)^†^
Black, non-Hispanic	**26,942**	**85.8 (85.0–86.5)**	87.2 (86.3–88.0)	79.1 (77.1–80.9)	1.10 (1.07–1.13)^†^
Asian/Pacific Islander, non-Hispanic	**7,336**	**89.8 (88.3–91.1)**	90.2 (88.4–91.7)	87.6 (84.9–89.8)	1.03 (1.00–1.06)
American Indian/Alaska Native, non-Hispanic	**2,771**	**84.1 (81.2–86.6)**	85.6 (82.2–88.5)	79.1 (73.2–84.0)	1.08 (1.00–1.17)^†^
Hispanic	**21,419**	**90.0 (89.2–90.6)**	90.8 (90.0–91.6)	84.9 (83.4–86.3)	1.07 (1.05–1.09)^†^
Other or multiple race, non-Hispanic	**7,131**	**87.2 (85.7–88.5)**	89.8 (88.3–91.2)	78.2 (74.6–81.4)	1.15 (1.10–1.20)^†^
**Age group (yrs)**					
18–24	**16,003**	**80.8 (79.8–81.8)**	82.6 (81.4–83.7)	74.7 (72.9–76.5)	1.11 (1.08–1.14)^†^
25–34	**28,728**	**84.5 (83.8–85.2)**	86.3 (85.5–87.1)	78.4 (77.0–79.7)	1.10 (1.08–1.12)^†^
35–44	**35,751**	**89.2 (88.6–89.7)**	90.8 (90.1–91.4)	83.7 (82.5–84.7)	1.09 (1.07–1.10)^†^
45–64	**114,304**	**90.3 (90.0–90.6)**	92.0 (91.6–92.3)	84.7 (84.1–85.3)	1.09 (1.08–1.10)^†^
≥65	**100,298**	**91.4 (91.0–91.7)**	92.8 (92.4–93.2)	86.6 (86.0–87.3)	1.07 (1.06–1.08)^†^
**Education**					
<High school	**20,900**	**85.7 (84.8–86.5)**	87.7 (86.7–88.7)	76.7 (74.8–78.5)	1.14 (1.11–1.18)^†^
High school	**76,320**	**85.7 (85.2–86.2)**	88.1 (87.5–88.6)	78.3 (77.4–79.2)	1.12 (1.11–1.14)^†^
Some college or greater	**200,515**	**89.7 (89.4–90.0)**	90.9 (90.6–91.2)	85.6 (85.1–86.1)	1.06 (1.06–1.07)^†^
**Marital status**					
Married	**157,360**	**90.8 (90.5–91.0)**	92.2 (91.9–92.5)	86.1 (85.6–86.6)	1.07 (1.06–1.08)^†^
Not married	**139,483**	**85.3 (84.9–85.7)**	87.2 (86.8–87.7)	78.7 (78.0–79.4)	1.11 (1.10–1.12)^†^
**Employment status**					
Currently employed	**149,514**	**87.6 (87.2–87.9)**	89.3 (88.9–89.6)	81.9 (81.3–82.5)	1.09 (1.08–1.10)^†^
Not employed	**147,844**	**88.8 (88.5–89.2)**	90.4 (90.0–90.8)	83.4 (82.8–84.1)	1.08 (1.07–1.09)^†^
**Body mass index**					
Underweight	**4,786**	**87.3 (85.2–89.2)**	87.9 (85.3–90.1)	85.2 (81.8–88.1)	1.03 (0.99–1.08)
Normal weight	**95,078**	**89.1 (88.7–89.5)**	90.4 (89.9–90.9)	84.9 (84.1–85.6)	1.06 (1.05–1.08)^†^
Overweight	**102,597**	**88.9 (88.6–89.3)**	90.6 (90.2–91.0)	83.3 (82.5–84.0)	1.09 (1.08–1.10)^†^
Obese	**81,019**	**85.6 (85.1–86.0)**	87.8 (87.2–88.3)	78.1 (77.2–79.0)	1.12 (1.11–1.14)^†^
**Alcohol-impaired driving **					
Yes	**4,100**	**73.7 (71.2–76.0)**	75.3 (72.3–78.1)	68.4 (64.2–72.3)	1.10 (1.03–1.18)^†^
No	**293,823**	**88.4 (88.2–88.6)**	90.0 (89.8–90.3)	82.8 (82.4–83.3)	1.09 (1.08–1.09)^†^
**Nonmetropolitan counties, overall**	**130,324**	**80.6 (80.1–81.0)**	**83.5 (82.9–84.0)**	**72.5 (71.7–73.3)**	**1.15 (1.14–1.17)^†^**
**Sex**					
Male	**53,540**	**74.4 (73.6–75.1)**	77.5 (76.6–78.4)	65.8 (64.5–67.1)	1.18 (1.15–1.21)^†^
Female	**76,784**	**86.4 (85.9–86.9)**	89.0 (88.4–89.6)	79.1 (78.1–80.1)	1.12 (1.11–1.14)^†^
**Race/Ethnicity**					
White, non-Hispanic	**110,991**	**79.9 (79.3–80.4)**	83.0 (82.4–83.6)	72.0 (71.1–72.9)	1.15 (1.14–1.17)^†^
Black, non-Hispanic	**5,160**	**82.7 (80.8–84.5)**	83.6 (81.6–85.4)	74.7 (67.9–80.5)	1.12 (1.03–1.12)^†^
Asian/Pacific Islander, non-Hispanic	**1,044**	**88.7 (82.6–92.8)**	90.3 (83.1–94.6)^§^	—^¶^	—
American Indian/Alaska Native, non-Hispanic	**3,759**	**80.9 (78.4–83.2)**	84.5 (81.3–87.2)	73.3 (69.3–77.1)	1.15 (1.08–1.23)^†^
Hispanic	**5,032**	**85.1 (82.9–87.0)**	87.1 (84.7–89.2)	76.4 (71.6–80.6)	1.14 (1.07–1.22)^†^
Other or multiple race, non-Hispanic	**2,708**	**82.3 (79.0–85.2)**	84.2 (80.2–87.6)	75.7 (70.0–80.5)	1.11 (1.03–1.21)^†^
**Age group (yrs)**					
18–24	**5,701**	**71.8 (69.7–73.7)**	75.1 (72.7–77.4)	63.3 (59.7–66.8)	1.19 (1.11–1.27)^†^
25–34	**10,735**	**74.9 (73.3–76.4)**	78.0 (76.1–79.9)	66.2 (63.5–68.7)	1.18 (1.13–1.24)^†^
35–44	**13,755**	**79.4 (78.0–80.6)**	82.2 (80.5–83.8)	71.3 (69.1–73.4)	1.15 (1.11–1.20)^†^
45–64	**51,225**	**83.0 (82.4–83.6)**	85.8 (85.0–86.5)	75.1 (73.9–76.2)	1.14 (1.12–1.16)^†^
≥65	**47,873**	**85.7 (85.2–86.3)**	88.3 (87.6–88.9)	78.6 (77.5–79.6)	1.12 (1.11–1.14)^†^
**Education**					
<High school	**11,647**	**78.4 (76.9–79.9)**	82.2 (80.5–83.8)	66.1 (62.9–69.2)	1.24 (1.18–1.31)^†^
High school	**44,323**	**79.1 (78.3–79.8)**	82.3 (81.4–83.2)	70.2 (68.9–71.6)	1.17 (1.15–1.20)^†^
Some college or greater	**74,086**	**82.5 (81.9–83.1)**	84.8 (84.1–85.5)	76.2 (75.3–77.1)	1.11 (1.10–1.13)^†^
**Marital status**					
Married	**72,908**	**82.3 (81.8–82.9)**	84.9 (84.3–85.6)	75.2 (74.2–76.1)	1.13 (1.11–1.15)^†^
Not married	**56,933**	**78.5 (77.7–79.2)**	81.7 (80.8–82.6)	69.1 (67.7–70.5)	1.18 (1.15–1.21)^†^
**Employment status**					
Currently employed	**63,218**	**77.5 (76.8–78.2)**	80.7 (79.8–81.5)	69.3 (68.1–70.4)	1.16 (1.14–1.19)^†^
Not employed	**66,601**	**84.1 (83.5–84.7)**	86.5 (85.8–87.2)	76.8 (75.6–77.9)	1.13 (1.11–1.15)^†^
**Body mass index**					
Underweight	**1,936**	**84.2 (80.6–87.2)**	85.4 (80.8–89.0)	80.7 (75.3–85.2)	1.06 (0.98–1.14)
Normal weight	**37,642**	**82.6 (81.7–83.5)**	85.4 (84.4–86.5)	75.1 (73.4–76.7)	1.14 (1.11–1.17)^†^
Overweight	**45,061**	**80.9 (80.1–81.7)**	83.4 (82.5–84.4)	73.8 (72.5–75.0)	1.13 (1.11–1.15)^†^
Obese	**39,457**	**77.8 (76.9–78.6)**	81.2 (80.2–82.2)	67.4 (65.8–68.9)	1.21 (1.17–1.24)^†^
**Alcohol-impaired driving**					
Yes	**1,696**	**60.6 (55.7–65.4)**	67.4 (60.7–73.4)	47.1 (40.3–54.1)	1.43 (1.20–1.70)^†^
No	**128,339**	**80.9 (80.4–81.4)**	83.7 (83.2–84.3)	73.0 (72.1–73.8)	1.15 (1.13–1.16)^†^

**Abbreviations:** CI = confidence interval; RR = rate ratio; RSE = relative standard error.

* In 2014, a total of 33 states and the District of Columbia (DC) had primary enforcement, 16 states had secondary enforcement, and one state (New Hampshire) had no requirement for seat belt use by adults. States with primary enforcement in 2014 included Alabama, Alaska, Arkansas, California, Connecticut, Delaware, DC, Florida, Georgia, Hawaii, Illinois, Indiana, Iowa, Kansas, Kentucky, Louisiana, Maine, Maryland, Michigan, Minnesota, Mississippi, New Jersey, New Mexico, New York, North Carolina, Oklahoma, Oregon, Rhode Island, South Carolina, Tennessee, Texas, Washington, West Virginia, and Wisconsin. States with secondary enforcement in 2014 included Arizona, Colorado, Idaho, Massachusetts, Missouri, Montana, Nebraska, Nevada, North Dakota, Ohio, Pennsylvania, South Dakota, Utah, Vermont, Virginia, and Wyoming. New Hampshire was grouped with secondary enforcement states for analysis purposes.

^†^ RRs comparing seat belt use in primary enforcement states to seat belt use in secondary enforcement states are significantly different from 1 (p<0.05).

^§^ Estimates based on 20–49 respondents and RSE <30% for rarer outcome (not always wearing seat belt) might be unreliable and should be interpreted with caution.

^¶^ Estimates based on <20 respondents or with RSE ≥30% for rarer outcome (not always wearing seat belt) were deemed unreliable and were suppressed.

For almost all demographic, health-related, and behavioral categories, seat belt use was significantly higher in primary enforcement states than in secondary enforcement states (Table 3). Seat belt use was 83.5% for residents of nonmetropolitan counties with primary enforcement, whereas seat belt use was significantly lower (72.5%) for residents of nonmetropolitan counties with secondary enforcement (RR = 1.15). In nonmetropolitan counties, some of the strongest associations by enforcement type were among those who had less than a high school education (RR = 1.24), were obese (RR = 1.21), and reported alcohol-impaired driving in the past 30 days (RR = 1.43).

## Discussion

Age-adjusted passenger-vehicle–occupant death rates increased with increasing rurality, with census region-specific age-adjusted death rates that were 3–10 times higher in the most rural counties than in the most urban counties. This trend also was found in the presence of both primary and secondary seat belt enforcement. This report provides additional information related to previously reported disparities in urban and rural crash deaths that have primarily used dichotomous measures to distinguish rural-urban areas (*1*,*5*,*9*). A single-state study that compared various measures of rurality, each with six levels on a rural-urban continuum, also found a consistent trend of higher death rates in increasingly rural counties (*12*).

A substantial predictor of a crash-related death among passenger vehicle occupants is lack of seat belt use (*6*). Lower use of seat belts in rural areas compared with urban areas is one factor that contributes to the increased risk for crash-related deaths in rural areas. This report indicates that self-reported seat belt use was highest in the most urban counties (88.8%) and lowest in the most rural counties (74.7%). This trend also was found in each of the four census regions of the United States and among both primary and secondary enforcement states. In addition, for almost all demographic, health-related, and behavioral characteristics examined, seat belt use was higher in metropolitan counties than in nonmetropolitan counties. These findings confirm and provide an update of multiple previous reports on rural-urban differences in seat belt use (*7*,*21*,*22*); however, the most recent National Occupant Protection Use Survey (NOPUS) daytime observational survey of drivers and front-seat passengers reported similar rates of seat belt use in rural (89.5%) and urban (90.5%) areas in 2016 (*23*). NOPUS methods exclude selected sites with very low traffic volume (*24*). Methodological factors, actual changes in the relationship between seat belt use and rural-urban status, or a combination of both might be responsible for the recent observational findings.

Seat belt use has been shown to prevent death from a motor-vehicle crash, and groups that are most at risk for nonuse can be the focus of interventions to increase seat belt use. Although overall levels of seat belt use in the United States are relatively high (86.9% in 2014), the small percentage who do not always wear seat belts represent a large proportion of the passenger-vehicle–occupant deaths. The 13% of adults who did not always wear seat belts represented 44.4% (in the most urban counties) to 61.3% (in the most rural counties) of adult passenger-vehicle–occupant deaths in 2014. Although some crashes are not survivable because of factors such as high-impact speed (*25*), seat belt use is one of the best ways a person can prevent serious injury or death in a crash. NHTSA estimates that an additional 2,814 deaths could have been prevented in 2014 if all passenger-vehicle occupants had been restrained (*26*).

Overall, and for each level of rural-urban designation, primary seat belt enforcement was associated with higher levels of seat belt use than secondary enforcement, confirming previous reports on seat belt use and rurality (*7*,*22*). For example, in the most rural counties, seat belt use was 79.1% in primary enforcement states compared with 64.7% in secondary enforcement states. Primary seat belt enforcement laws and high-visibility enforcement of seat belt laws also have been associated with reduced numbers of crash deaths (*10*,*11*). A study that examined the effect of enforcement type on passenger-vehicle–occupant death rates during 2001–2010 concluded that adjusted death rates were 17% lower in primary enforcement states than in secondary enforcement states (*11*). One study found no significant effect of seat belt enforcement type on passenger-vehicle–occupant death rates after controlling for secular trends and fixed state effects (*27*).

Of the four census regions examined in this report, the South had the highest age-adjusted passenger-vehicle–occupant death rate overall (10.7 passenger-vehicle–occupant deaths per 100,000 population) and in both metropolitan and nonmetropolitan areas. One study of police-reported crashes found that passenger-vehicle occupants who lived in the South were more likely to experience severe or fatal injuries than those who did not live in the South, even after controlling for factors such as restraint use, high vehicle speed, and rurality (*28*). In addition to high age-adjusted passenger-vehicle–occupant death rates for the South overall, only one southern state (Virginia) had a secondary enforcement law, and Virginia has one of the lowest death rates from motor-vehicle crashes (8.4 total crash-related deaths per 100,000 population in 2014) among all secondary enforcement states (range: 4.9 to 25.7 deaths per 100,000 population) (*29*). The high age-adjusted passenger-vehicle–occupant death rates in the South and the relatively low overall death rate from motor-vehicle crashes in Virginia compared with other secondary enforcement states might explain the observed relationship between age-adjusted passenger-vehicle–occupant death rates and enforcement type (primary or secondary) in the South. Age-adjusted passenger-vehicle–occupant death rates were higher in secondary enforcement states than in primary enforcement states for each of the other three census regions.

Other factors contribute to the higher death rates in rural areas. For example, high speed (whether indicated by a high posted road speed limit or a high travel speed at the time of crash) is associated with increased risk for death in motor-vehicle crashes and is more common in fatal crashes in rural areas (*28*,*30*). Rollover crashes are more common in rural areas (*1*). Roadway characteristics (e.g., lack of paved road shoulders) that are more common in rural areas might increase the likelihood and severity of crashes (*31*). A Nebraska study found increased odds of crash-related deaths in rural areas, even after controlling for injury severity, suggesting that rural-urban differences in medical care might affect risk for death (*32*). Prehospital and hospital care indicators, such as shorter transport times (from crash scene to hospital) and the presence of a trauma center (level I–IV), have been associated with lower rates of death following a motor-vehicle crash, and rural areas are less likely than urban areas to have such resources (*33*). Rural areas also have higher proportions of older adults (≥65 years) (*34*), and older adults are at increased risk for severe injury and death after a crash because of various factors such as frailty (*5*,*35*,*36*).

## Limitations

The findings in this report are subject to at least five limitations. First, although crash deaths were coded geographically by county of crash location, the population data used to calculate age-adjusted death rates were coded by county of residence. Bias in rates could occur in either direction to the extent that fatal crash and residence locations differ. A statewide survey found that approximately three fourths of trips were to locations of the same rural-urban designation (using a six-level rural-urban designation based on zip codes) as respondents’ place of residence (*12*). When travel crossed rural-urban designations, rural residents more likely traveled to urban areas than the opposite, suggesting that any bias would be to underestimate the rural-urban disparity in age-adjusted death rates (*12*). Second, Virginia was the only state in the southern region with a secondary enforcement law and might not be a truly representative comparison group in the South. Among secondary enforcement states, Virginia had a relatively low age-adjusted passenger-vehicle–occupant death rate (7.0 per 100,000), despite being in a census region with a high passenger-vehicle–occupant death rate. Third, other factors associated with crash-related deaths, seat belt use, or both (e.g., alcohol-impaired driving and speeding) were not controlled for in the analysis and could have affected the associations between state seat belt enforcement and age-adjusted passenger-vehicle–occupant death rates or seat belt use. Fourth, BRFSS seat belt use data were self-reported by respondents and might be subject to social desirability bias. However, a comparison of self-reported and observational seat belt use in 2008 found minimal bias in self-reported estimates (*37*). Finally, the median response rate for the 2014 BRFSS was 47%; nonresponses might have differed across rural-urban designations. 

## Future Directions

Reducing crash-related deaths in rural areas requires a multifaceted approach. Implementation of effective interventions, including primary enforcement seat belt laws and high-visibility enforcement (i.e., increased police enforcement efforts coupled with highly publicized media campaigns), can increase seat belt use and reduce the likelihood of severe injuries and deaths (*10*). CDC has developed several resources and tools that states and communities can use to identify effective interventions that might help to address rural-urban disparities in seat belt use and passenger-vehicle–occupant death rates. These include the Motor Vehicle Prioritizing Interventions and Cost Calculator for States (MV PICCS), which calculates the expected number and monetized value of injuries prevented and lives saved at the state level after implementation of up to 14 proven strategies (https://www.cdc.gov/motorvehiclesafety/calculator), and *The* *Guide to Community Preventive Services* (e.g., The Community Guide), a collection of systematic reviews of evidence-based findings of the Community Preventive Services Task Force that includes motor-vehicle injury prevention reviews (https://www.thecommunityguide.org). In addition, NHTSA’s Countermeasures that Work is a reference to assist states in the selection of effective, evidence-based countermeasures for traffic safety (https://www.nhtsa.gov/sites/nhtsa.dot.gov/files/812202-countermeasuresthatwork8th.pdf). Finally, future studies using multivariate models could be conducted to adjust for effects confounding the association between seat belt enforcement type, rurality, and passenger-vehicle–occupant deaths. This future research into the effects of rurality and census region on passenger vehicle occupant safety will continue to be used for efforts to reduce rural-urban disparities in crash-related risk behaviors, injuries, and deaths.

## Conclusion

Seat belt use prevented an estimated 64,000 deaths in the United States during 2011–2015 (*38*). Although self-reported levels of seat belt use reached 86.9% in 2014, a marked increase in recent decades, the small percentage who still do not always use seat belts represent almost half of all occupant deaths in the United States, and rural residents are disproportionately affected. Despite differences in how rural and urban areas are defined in various studies, rurality is consistently shown to be associated with increased crash-related death rates and lower seat belt use. Improving seat belt use remains a critical strategy to reduce crash-related deaths in the United States, especially in rural areas where seat belt use is lower and age-adjusted passenger-vehicle–occupant death rates are higher than in urban areas.

## References

[1] National Highway Traffic Safety Administration. Traffic safety facts 2015 data: rural/urban comparison of traffic fatalities. Washington, DC: National Highway Traffic Safety Administration; 2017. https://crashstats.nhtsa.dot.gov/Api/Public/Publication/812393

[2] CDC. QuickStats: age-adjusted rate of motor vehicle traffic deaths, by urbanization of county of residence—2005 and 2015. MMWR Morb Mortal Wkly Rep 2017;66:567. 10.15585/mmwr.mm6621a6 28570502 PMC5657821

[3] US Census Bureau. Measuring America: our changing landscape. Washington, DC: US Census Bureau; 2016. https://www.census.gov/library/visualizations/2016/comm/acs-rural-urban.html

[4] National Highway Traffic Safety Administration. Traffic safety facts 2015 data: passenger vehicles. Washington, DC: National Highway Traffic Safety Administration; 2017. https://crashstats.nhtsa.dot.gov/Api/Public/ViewPublication/812413

[5] Zwerling C, Peek-Asa C, Whitten PS, Choi SW, Sprince NL, Jones MP. Fatal motor vehicle crashes in rural and urban areas: decomposing rates into contributing factors. Inj Prev 2005;11:24–8. 10.1136/ip.2004.005959 15691985 PMC1730169

[6] National Highway Traffic Safety Administration. Fourth report to Congress: effectiveness of occupant protection systems and their use. Washington, DC: US Department of Transportation; 1999.

[7] Strine TW, Beck LF, Bolen J, Okoro C, Dhingra S, Balluz L. Geographic and sociodemographic variation in self-reported seat belt use in the United States. Accid Anal Prev 2010;42:1066–71. 10.1016/j.aap.2009.12.014 20441814

[8] CDC. Vital signs: nonfatal, motor vehicle—occupant injuries (2009) and seat belt use (2008) among adults—United States. MMWR Morb Mortal Wkly Rep 2011;59:1681–6. 21209609

[9] Ash IK, Edwards AL, Porter BE. An investigation of state population characteristics that moderate the relationship of state seat belt law and use in the United States. Accid Anal Prev 2014;71:129–36. 10.1016/j.aap.2014.05.011 24915200

[10] Dinh-Zarr TB, Sleet DA, Shults RA, ; Task Force on Community Preventive Services. Reviews of evidence regarding interventions to increase the use of safety belts. Am J Prev Med 2001;21(Suppl):48–65. 10.1016/S0749-3797(01)00378-6 11691561

[11] Lee LK, Monuteaux MC, Burghardt LC, Motor vehicle crash fatalities in states with primary versus secondary seat belt laws: a time-series analysis. Ann Intern Med 2015;163:184–90. 10.7326/M14-2368 26098590

[12] McAndrews C, Beyer K, Guse CE, Layde P. How do the definitions of urban and rural matter for transportation safety? Re-interpreting transportation fatalities as an outcome of regional development processes. Accid Anal Prev 2016;97:231–41. 10.1016/j.aap.2016.09.008 27693862

[13] US Department of Agriculture [Internet]. Rural-urban continuum codes. Washington, DC: US Department of Agriculture; 2016. https://www.ers.usda.gov/data-products/rural-urban-continuum-codes

[14] Ball CG, Kirkpatrick AW, Brenneman FD. Noncompliance with seat-belt use in patients involved in motor vehicle collisions. Can J Surg 2005;48:367–72. 16248134 PMC3211890

[15] National Highway Traffic Safety Administration. Fatality analysis reporting system (FARS): analytical user’s manual 1975–2015. Washington, DC: National Highway Traffic Safety Administration; 2016. https://crashstats.nhtsa.dot.gov/Api/Public/Publication/812315

[16] National Center for Health Statistics, CDC. Documentation for vintage 2015 bridged-race postcensal population estimates for calculating vital rates. Washington, DC: National Center for Health Statistics, CDC, US Department of Health and Human Services; 2016. https://www.cdc.gov/nchs/nvss/bridged_race/documentation_bridged_postcenv2015.pdf

[17] Dwyer-Lindgren L, Bertozzi-Villa A, Stubbs RW, U.S. county-level trends in mortality rates for major causes of death, 1980–2014. JAMA 2016;316:2385–401. 10.1001/jama.2016.13645 27959996 PMC5576343

[18] Insurance Institute for Highway Safety [Internet]. Arlington, VA: Insurance Institute for Highway Safety. http://www.iihs.org

[19] US Census Bureau. Census regions and divisions of the United States. Washington, DC: US Census Bureau. https://www2.census.gov/geo/pdfs/maps-data/maps/reference/us_regdiv.pdf

[20] National Highway Traffic Safety Administration. Summary of vehicle occupant protection and motorcycle laws. Washington, DC: National Highway Traffic Safety Administration; 2015. https://www.nhtsa.gov/staticfiles/nti/pdf/812129-SummaryVehicleOccupantProtection-MotorcycleLaws.pdf

[21] National Highway Traffic Safety Administration. Seat belt use in 2014–overall results. Washington, DC: National Highway Traffic Safety Administration; 2015. https://crashstats.nhtsa.dot.gov/Api/Public/ViewPublication/812113

[22] Sunshine J, Dwyer-Lindgren L, Chen A, Mokdad AH. Seat-belt use in U.S. counties: limited progress toward Healthy People 2020 objectives. Health Aff (Millwood) 2017;36:636–9. 10.1377/hlthaff.2016.1345 28373328

[23] National Highway Traffic Safety Administration. Seat belt use in 2016–overall results. Washington, DC: National Highway Traffic Safety Administration; 2016. https://crashstats.nhtsa.dot.gov/Api/Public/ViewPublication/812351

[24] National Highway Traffic Safety Administration. Occupant restraint use in 2015: results from the NOPUS controlled intersection study. Washington, DC: National Highway Traffic Safety Administration; 2016. https://crashstats.nhtsa.dot.gov/Api/Public/ViewPublication/812330

[25] Sobhani A, Young W, Logan D, Bahrololoom S. A kinetic energy model of two-vehicle crash injury severity. Accid Anal Prev 2011;43:741–54. 10.1016/j.aap.2010.10.021 21376862

[26] National Highway Traffic Safety Administration. Lives saved in 2014 by restraint use and minimum-drinking-age laws. Washington, DC: National Highway Traffic Safety Administration; 2015. https://crashstats.nhtsa.dot.gov/Api/Public/ViewPublication/812218

[27] Harper S, Strumpf EC. Primary enforcement of mandatory seat belt laws and motor vehicle crash deaths. Am J Prev Med 2017;53:176–83. 10.1016/j.amepre.2017.02.003 28336356

[28] Travis LL, Clark DE, Haskins AE, Kilch JA. Mortality in rural locations after severe injuries from motor vehicle crashes. J Safety Res 2012;43:375–80. 10.1016/j.jsr.2012.10.004 23206510 PMC3514883

[29] National Highway Traffic Safety Administration. Traffic safety facts 2014 data—state traffic data. Washington, DC: National Highway Traffic Safety Administration; 2016. https://crashstats.nhtsa.dot.gov/Api/Public/Publication/812293

[30] Donaldson AE, Cook LJ, Hutchings CB, Dean JM. Crossing county lines: the impact of crash location and driver’s residence on motor vehicle crash fatality. Accid Anal Prev 2006;38:723–7. 10.1016/j.aap.2006.01.002 16480940

[31] Federal Highway Administration. Manual for selecting safety improvements on high risk rural roads. Washington, DC: Federal Highway Administration; 2014. https://safety.fhwa.dot.gov/hsip/hrrr/manual/hrrr_2014.pdf

[32] Muelleman RL, Wadman MC, Tran TP, Ullrich F, Anderson JR. Rural motor vehicle crash risk of death is higher after controlling for injury severity. J Trauma 2007;62:221–5, discussion 225–6. 10.1097/01.ta.0000231696.65548.06 17215759

[33] Melton SM, McGwin G Jr, Abernathy JH 3rd, MacLennan P, Cross JM, Rue LW 3rd. Motor vehicle crash-related mortality is associated with prehospital and hospital-based resource availability. J Trauma 2003;54:273–9. 10.1097/01.TA.0000038506.54819.11 12579051

[34] US Census Bureau. 65+ in the United States: 2010. Washington, DC: US Government Printing Office; 2014. https://www.census.gov/content/dam/Census/library/publications/2014/demo/p23-212.pdf

[35] Li G, Braver ER, Chen LH. Fragility versus excessive crash involvement as determinants of high death rates per vehicle-mile of travel among older drivers. Accid Anal Prev 2003;35:227–35. 10.1016/S0001-4575(01)00107-5 12504143

[36] Carter PM, Flannagan CA, Reed MP, Cunningham RM, Rupp JD. Comparing the effects of age, BMI and gender on severe injury (AIS 3+) in motor-vehicle crashes. Accid Anal Prev 2014;72:146–60. 10.1016/j.aap.2014.05.024 25061920 PMC4753843

[37] Ibrahimova A, Shults RA, Beck LF. Comparison of 2008 national and state-level self-reported and observed seatbelt use estimates. Inj Prev 2011;17:201–3. 10.1136/ip.2010.028597 21393414

[38] National Highway Traffic Safety Administration. Lives saved in 2015 by restraint use and minimum-drinking-age laws. Washington, DC: National Highway Traffic Safety Administration; 2016. https://crashstats.nhtsa.dot.gov/Api/Public/ViewPublication/812319

